# Clinical improvement of encapsulating peritoneal sclerosis after challenging course and 6 months of total parenteral nutrition in child with nephronophthisis: a case report

**DOI:** 10.1186/s13256-021-02905-3

**Published:** 2021-07-13

**Authors:** Saeed Mohammed AlZabali, Abdulkarim AlAnazi, Khawla A. Rahim, Hassan Y. Faqeehi

**Affiliations:** Pediatric Nephrology Section, Children Hospital, King Fahad Medical City, Riyadh, Saudi Arabia

**Keywords:** Peritoneal sclerosis, Recovery, Peritonitis, Refractory

## Abstract

**Background:**

Encapsulating peritoneal sclerosis is a rare but potentially lethal complication of long-term peritoneal dialysis that is associated with significant morbidity and mortality. The occurrence of encapsulating peritoneal sclerosis varies worldwide, but is increased in patients maintained on peritoneal dialysis for 5–8 years. The etiology of encapsulating peritoneal sclerosis remains unidentified, and a high index of clinical suspicion is required for diagnosis.

**Case presentation:**

We report a 5-year-old Saudi female with end-stage renal disease secondary to nephronophthisis type 2. She underwent peritoneal dialysis for 30 months, with four episodes of peritonitis. She presented with clinical signs of peritonitis. Three days later, she developed septic shock, which required pediatric intensive care unit admission. The peritoneal dialysis catheter was removed because of refractory peritonitis. Her course was complicated by small bowel perforation, and severe adhesions were revealed on abdominal ultrasound and computed tomography, consistent with a diagnosis of EPS. This finding was later confirmed by diagnostic laparotomy performed twice and complicated by recurrent abdominal wall fistula. She received total parenteral nutrition for 6 months and several courses of antibiotics. The patient received supportive treatment including nutritional optimization and treatment for infection. No other treatments, such as immunosuppression, were administered to avoid risk of infection. Following a complicated hospital course, the patient restarted oral intake after 6 months of total parenteral nutrition dependency. Her abdominal fistula resolved completely, and she was maintained on hemodialysis for few years before she received a kidney transplant.

**Conclusion:**

When treating patients using peritoneal dialysis, it is important to consider encapsulating peritoneal sclerosis with refractory peritonitis, which is not always easy to identify, particularly if the patient has been maintained on peritoneal dialysis for less than 3 years. Early identification of encapsulating peritoneal sclerosis and appropriate conservative treatment, including nutritional optimization and treatment of infections, are essential to achieve a better prognosis.

## Background

Peritoneal dialysis (PD) is a successful dialysis modality that enables patients with end-stage kidney disease to receive a home-based treatment. It has many advantages for patient quality of life, and the survival outcomes of PD are equal to those of hemodialysis (HD) [1]. Encapsulating peritoneal sclerosis (EPS) is a potential, although extremely rare, complication of long-term PD, and is defined by the International Society of Peritoneal Dialysis as “a clinical syndrome continuously, intermittently or repeatedly presenting with symptoms of intestinal obstruction due to adhesions of a diffusely thickened peritoneum” [2].

Diagnosis of EPS is based on a combination of bowel obstruction and features of encapsulation due to peritoneal fibrosis [1]. It differs from simple peritoneal fibrosis or thickened peritoneal membrane without the presence of obstruction/encapsulation symptoms [3]. It is normally a two-hit phenomenon, where the first hit is long-term PD and the second is any of the precipitating agents [3] .The reported mortality rate of EPS is high at around 35–69% [4], and is usually related to bowel obstruction and malnutrition [1]. The prevalence of EPS varies between 0.4% and 8.9%, its incidence rate is between 0.7 and 13.6 per 1000 patient-years, and its risk of occurrence after 5 years on PD is between 0.6% and 6.6% [1]. A high index of suspicion is required for an early diagnosis, and a prompt diagnosis and management may influence outcome [5]. It was observed first in 1907 and was called peritonitis chronica fibrosa encapsulate [1]. EPS was also reported by Gandhi *et al*. in 1980 and is characterized by a progressive intraabdominal inflammatory process that results in sheets of fibrous tissue [1]. The pathophysiology of EPS remains unclear, but it is likely to be multifactorial [6]. The symptoms of EPS range from mild complaints, such as abdominal pain, abdominal fullness, vomiting, constipation, or diarrhea, to complete intestinal obstruction. Unfortunately, these symptoms are not specific to EPS, and many patients have a history of gastrointestinal symptoms over long periods of time before a diagnosis is made [6]. Nakamoto suggested that EPS should be classified into four stages: pre-EPS, inflammatory, encapsulating, and obstructive [7]. Markers of an inflammatory state including elevated C-reactive protein, anemia, and hypoalbuminemia may be present, but there are no specific diagnostic effluent or blood markers for EPS [3]. The most specific findings using computed tomography (CT) are reported to be bowel tethering and peritoneal calcification, with additional CT findings of bowel wall thickening and dilatation secondary to obstruction [8]. Mild peritoneal membrane changes on CT without encapsulation or gastrointestinal dysfunction are not sufficient for a diagnosis of EPS [3]. Most studies have consistently identified longer PD duration as a key risk factor for the development of EPS [9–12]. Other parameters that have been identified in at least one study as possible risk factors of EPS include higher dialysate glucose exposure, use of conventional PD solutions (as opposed to biocompatible PD solutions), peritonitis (frequent, severe, or prolonged), younger age (likely due to lower competing risk of death), abdominal surgery, β-blocker use, icodextrin use, kidney transplantation, ultrafiltration (UF) failure, and higher peritoneal solute transport rate [1, 9, 11, 13]. EPS presents after withdrawal from PD in the majority (70–90% in some series) of cases [14]. The period between PD cessation and the development of EPS has been reported to last as long as 5 years [15]. There is no clear recommendation for medical therapy for treating the inflammatory and fibrotic features of EPS [3]. Therefore, appropriate nutritional support is imperative in the management of these patients [16].

## Case presentation

We report a 5-year-old, Saudi girl with end-stage renal disease secondary to nephronophthisis type 2, treated with automated peritoneal dialysis (APD) and chronic kidney disease medications since the age of 2 years. There was no family history of the same disease. She experienced four episodes of peritonitis. In the last episode, she presented to emergency room with vomiting and diarrhea that had persisted for a few days. She was dehydrated, not in respiratory distress, afebrile, blood pressure of 95/50 mmHg, and heart rate of 110 beats per minute, and initial biochemistry analysis showed hemoglobin of 12 g/dL, white blood cell (WBC) count of 6.3 × 10^9^/L, creatinine of 575 μmol/L, and urea of 20 mmol/L.

PD fluid analysis showed WBC count of 2736/mm^2^ and neutrophils of 93%, and culture confirmed *Staphylococcus* peritonitis. The patient was initially started on intraperitoneal ceftazidime with vancomycin. One day later, her condition deteriorated and she developed septic shock and was transferred to the pediatric intensive care unit. The PD catheter was removed and switched to continuous renal replacement therapy, and she was maintained on intermittent HD.

**Fig. 1:**
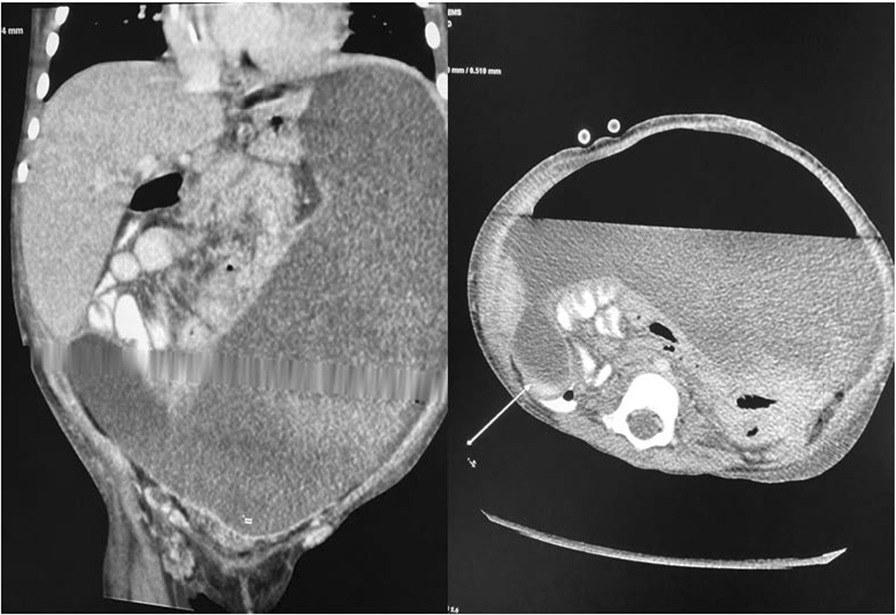
CT scan showing huge fluid collection in the abdomen and large air fluid level with peritoneal thickening. The arrow is pointing to peritoneal thickening

**Fig. 2:**
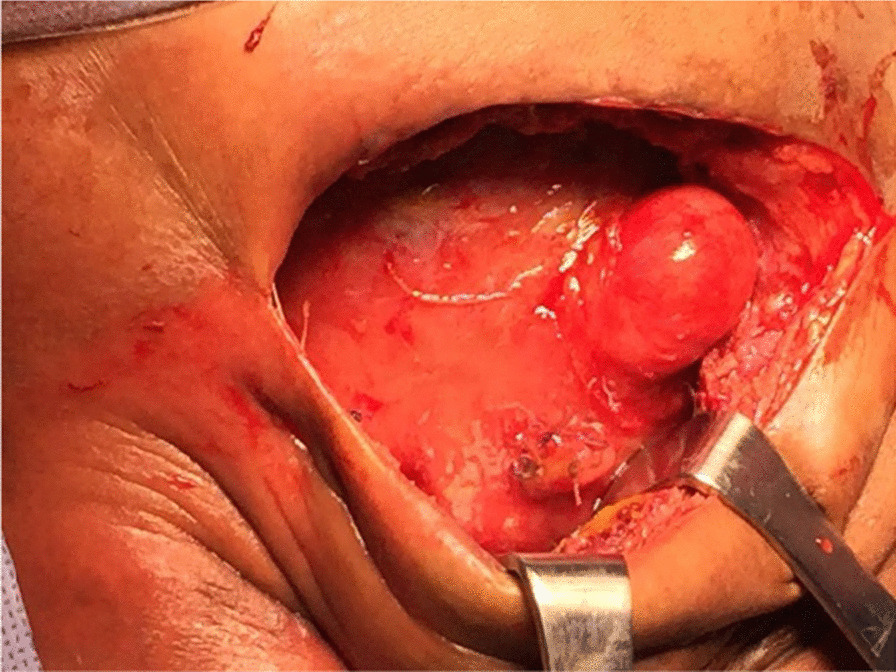
Severe adhesion seen during laparotomy

**Fig. 3:**
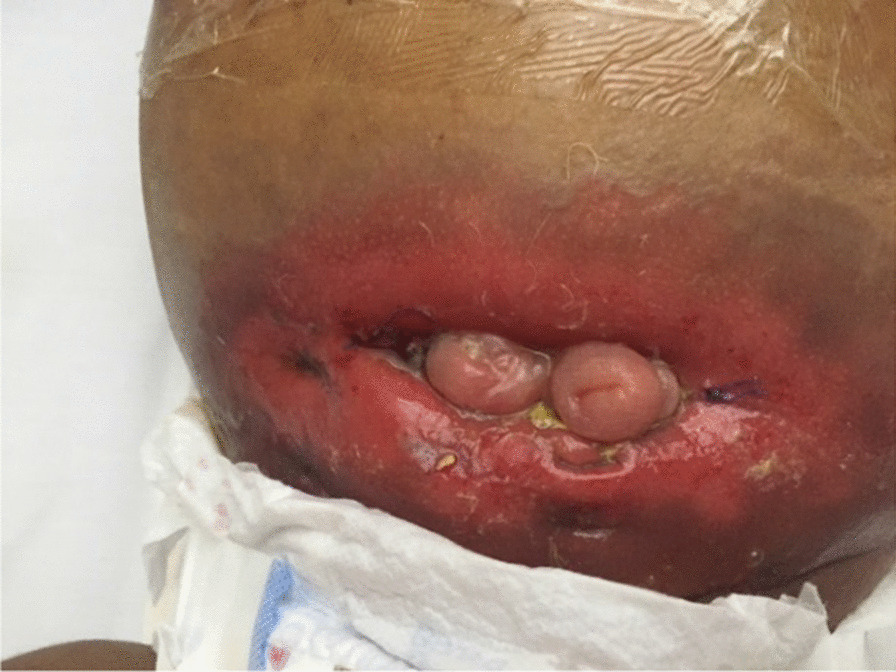
Proximal and distal stomas

**Fig. 4:**
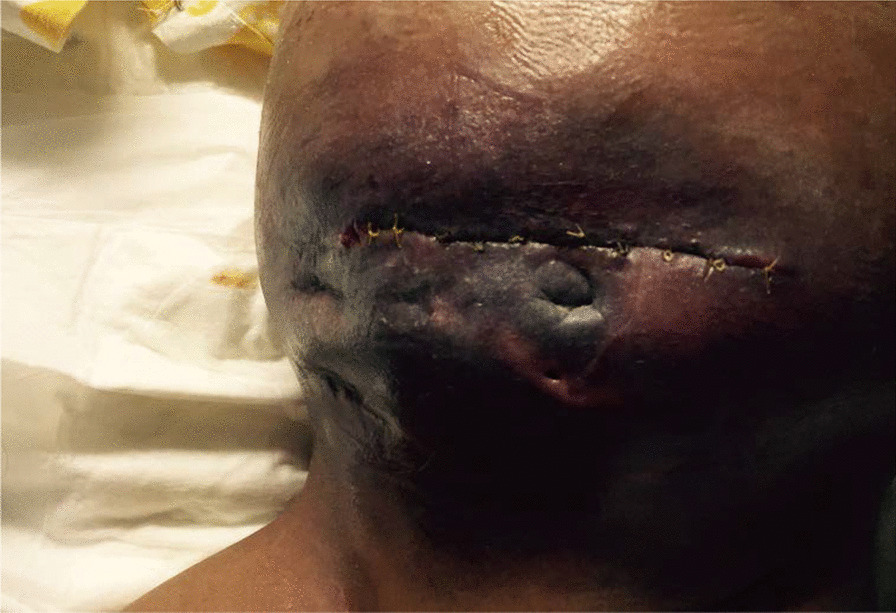
Recurrent fistula after stoma closure

**Fig. 5:**
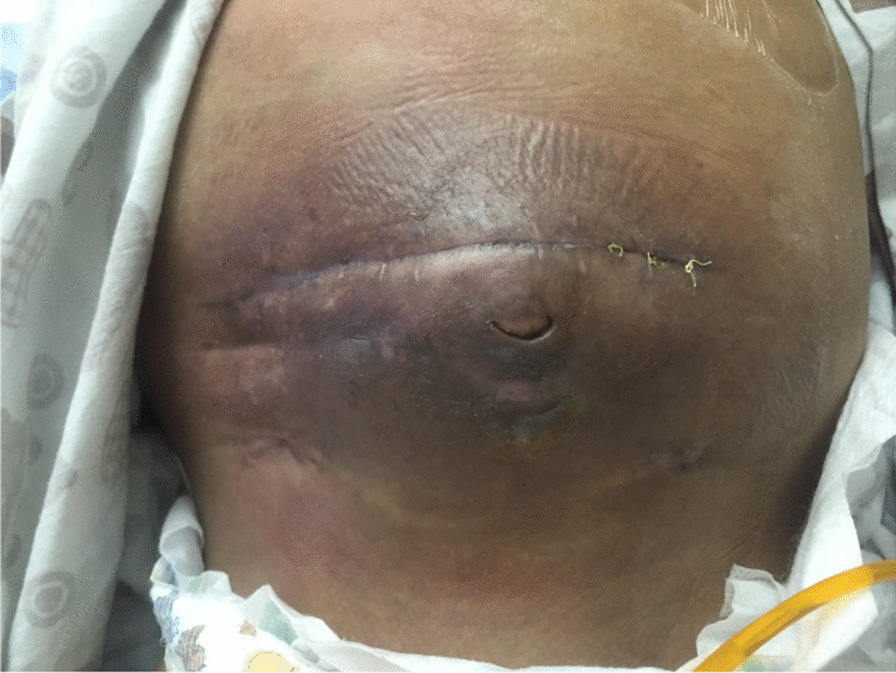
Healing of fistula

Plain abdominal X-ray revealed abnormal air lucency around the center of the abdomen, suggesting pneumoperitoneum. Abdominal CT findings revealed a huge fluid collection in the abdomen with a large air fluid level, extending from the level of the left hemidiaphragm to the pelvis. Mild enhancement of the peritoneum was noted with peritoneal thickening in the right lower quadrant (Fig. 1). The fluid collection displaced the abdominal viscera superiorly and posteriorly. Mild thickening of the right lateral abdominal wall was noted below the inferior margin of the liver, consistent with EPS. Ultrasound-guided abdominal drain aspirated 500 mL of pus mixed with fecal matter, likely due to bowel perforation. Laparotomy findings included extensive adhesions, fecal fluid, and bowel perforation. The whole bowel was matted together covered by thick membrane; the membrane was densely adherent to the bowel wall, which was difficult to separate (Fig. 2). Therefore, peritoneal biopsy was not done during surgery. An abdominal drain was inserted. A stoma was required but was difficult to create because of matted bowel. Two months later, a stoma was created but complicated by fistula with huge secretion that improved with conservative treatment (Figs. 3, 4). During hospitalization, the patient required a prolonged course of total parenteral nutrition (TPN) for 6 months until the gut healed and due to several instances of sepsis (Fig. 5). The patient made a complete recovery and remained stable on regular HD treatment and gained muscle mass. The child was on hemodialysis for 3 years before getting renal transplant from a cadaveric donor. Two and a half years post renal transplant, she is doing well on triple immunosuppressants (prednisolone, tacrolimus, and mycophenolate malate) without recurrence of EPS.

## Discussion and conclusion

Chronic PD can be complicated by EPS, which is considered the most severe complication associated with long-term PD [12]. It is not clear why some individuals develop EPS, but chronic exposure of the peritoneum to PD fluids is thought to provoke activation of various inflammatory, fibrogenic, and angiogenic cytokines that eventually lead to progressive peritoneal fibrosis, vasculopathy, and calcification [17]. There is growing interest in this area in adult CPD patients, particularly in the incidence, risk factors, treatment, and outcome of EPS [12, 18]. Due to the rarity of EPS, there are few reports of end-stage renal failure in children receiving CPD, and most evidence is anecdotal [19]. Some recommend that patients with impaired peritoneal ultrafiltration or peritoneal calcification on PD for more than 5 years should be carefully managed as presumptive cases of sclerosing encapsulating peritonitis (SEP) [20]. Some studies have shown that longer duration of PD is associated with a higher incidence of SEP. Therefore, the duration of PD is considered an important risk factor for SEP [20]. Araki *et al*. performed peritoneal biopsies in 14 children who had received PD for more than 5 years and found that 6 had simple sclerosis, while the others showed severe peritoneal sclerosis. The authors concluded that peritoneal biopsy should be performed if a patient receives PD for more than 5 years with poor UF and peritoneal calcifications on computed tomography scan [21]. Our case developed EPS after receiving PD for less than 3 years. Infections and growth failure are major problems in pediatric patients on chronic PD and have been extensively studied, while few data are available concerning the occurrence of EPS in this age group. Previous studies have suggested that PD-related peritonitis may predispose to EPS, mostly if is recurrent or caused by *Staphylococcus aureus*, fungi, or *Pseudomonas aeruginosa* [22]. The number of times a patient had peritonitis did not seem to represent itself a risk factor for EPS, whereas a single severe acute episode of peritonitis may be sufficient to activate the EPS pathophysiological cascade in an already impaired peritoneal membrane [8]. The European Paediatric Dialysis Working Group (EPDWG) study found that children with EPS had peritonitis episodes more frequently than all other PD patients [5], but this association was not found in an Italian study [12] in which the overall incidence of peritonitis was not significantly different from that in children from the same registry who did not develop EPS [6]. Our patient experienced several cases of peritonitis while she received PD, which could be a risk factor for EPS. Historically, the type of PD solution used is another important factor for EPS [6]. Nakao *et al*. concluded that use of biocompatible PD fluid contributed to a decrease in EPS development [23]. UF failure is mainly caused by a large vascular surface of peritoneal membrane that leads to an increased permeability (dialysate-to-creatinine plasma ratio > 0.80) and faster glucose absorption. This can result in a requirement for high dialysate glucose concentrations that can further worsen both the peritoneal membrane structure and function [24]. Interestingly, the EPDWG and Italian studies found no difference in EPS prevalence based on exposure to conventional compared with newer “biocompatible” dialysate, which is bicarbonate-based and has a lower glucose degradation product (GDP) [5, 12]. EPS may occur during treatment with CPD after a patient switches to HD or following kidney transplantation. Posttransplantation EPS occurs with increasing frequency in adults [12, 25]. The Japanese Ministry of Welfare working group proposed steroids as a potential therapy for EPS, leading to their widespread use in Japan. A report from one unit in Japan suggested an improvement in survival following introduction of steroids for treatment of EPS [26]. A large recent UK series showed no difference in outcomes for patients treated with steroids,immunosuppressants, tamoxifen, or combinations of these compared with patients who did not receive treatment [4]. Any beneficial effects of steroids are most likely to occur at an earlier, inflammatory stage of the disease [6]. There is interest in the use of tamoxifen in EPS based on its effectiveness in other fibrotic conditions as well as case reports and small series suggesting its therapeutic benefits in EPS. Tamoxifen avoids the catabolic and immunosuppressive effects of corticosteroids and other immunosuppressant in conditions in which malnutrition and sepsis are important determinants of mortality [27, 28]. We were unable to use immunosuppressive medication in our case because of recurrent infection during the hard treatment course, and the only management was surgical intervention and optimization of nutritional status by TPN that lasted around 6 months. The rationality for not giving tamoxifen is that there is no strong evidence to give tamoxifen in EPS , particularly in pediatric patients .In experienced hands, surgery results in high rates of improvement in symptoms and survival. Surgery should be performed in a setting in which the surgical team has experience with and understands the condition, and has appropriate perioperative renal and intensive care support [3]. In severe cases, and cases with degrees of bowel obstruction, parenteral nutrition is usually essential [3].

In conclusion, SEP is a rare but severe complication of PD. A high index of suspicion is required to diagnose EPS earlier. Malnutrition is a major problem in patients with severe EPS, and appropriate nutritional support with enteral or parenteral nutrition is required. Surgery is important in the management of SEP as well as optimization of growth and treating infections to achieve better prognosis.

## Data Availability

Not applicable.

## Abbreviations

APD: Automated peritoneal dialysis
CT: Computed tomography
EPDWG: European Paediatric Dialysis Working Group
EPS: Encapsulating peritoneal sclerosis
PD: Peritoneal dialysis
SEP: Sclerosing encapsulating peritonitis
TPN: Total parenteral nutrition
WBC: White blood cell
UF: Ultrafiltration failure
HD: Hemodialysis
PICU: Pediatric intensive care unit
